# Data exploration on factors that influences construction cost and time performance on construction project sites

**DOI:** 10.1016/j.dib.2018.02.035

**Published:** 2018-02-16

**Authors:** Lekan M. Amusan, Adedeji Afolabi, Raphael Ojelabi, Ignatius Omuh, Hilary I. Okagbue

**Affiliations:** a Department of Building Technology, Covenant University, Ota, Nigeria; b Department of Mathematics, Covenant University, Ota, Nigeria

**Keywords:** Likert scale, Questionnaire, Construction, Cost overrun, Severity index, Survey, Statistics

## Abstract

This data article explores the factors that contribute to maintaining steady cost projection on construction projects. The data was obtained using structured questionnaire designed in Likert scale. The responses were solicited from category of construction practitioners. Simple random sampling was employed in the distribution of the questionnaires to the respondents. Data samples were analysed using severity index, ranking and simple percentages. The analysis of the data brought to fore some important data on factors that causes cost overrun, they include: contractor's inexperience, inadequate planning, inflation, incessant variation order, and change in project design. They are critical to causing cost overrun, while project complexity, shortening of project period and fraudulent practices are found to be responsible. The data fall within the percentages of possible consequences of cost overrun when compared with those available in scientific literature. The data can provide insights on how to mitigate the risks of project deviation from initial cost and as-built project.

**Specifications Table**

**Table:** 

Subject area	Building Construction
More specific subject area	Construction Management
Type of data	Table, text file.
How data was acquired	Field survey
Data format	Raw, filtered and analyzed data
Experimental factors	Simple percentages and severity index were used as analytical tool of the generated data. SPSS (Statistical Packages for Social Science Students) was used in determining the nature, strength and pattern of relationships among the cost determinants and variables. The factors were ranked in order of their degree of severity.
Experimental features	The key method used in data collection structured questionnaire designed in Likert scale, the questionnaire was designed in such a way that it helps to collate basic information from the respondents. A population size of seventy (70) was selected, and a total sample size of 59 respondents was used in data generation, with questionnaire distributed to construction professionals. Variables pertaining to the above listed targets were identified and incorporated into questionnaires as the primary source of data. The data was collated and analysed, using mean item score ranking, percentages and descriptive statistics.
Data source location	Covenant University, Ota, Nigeria
Data accessibility	The article is in public repository http://eprints.covenantuniversity.edu.ng/

**Value of the data**

i. The data is useful in research that involves studying cost performance of construction projects.
ii. Data presented is useful in studying cost overrun that would help client and professional in project cost planning.
iii. The data could be used in development of cost and time models.
iv. The data is valuable to construction project professionals and could be used in policy formulation.
v. The data could be used as basis of comparison with that of other countries in terms of project management.

## 1 Data

The data was obtained using structured questionnaire designed in Likert scale. The responses were solicited from category of 70 construction practitioners using survey sampling methodology. The data retrieved from the 70 practitioners are presented as follows: data of professional affiliation of respondents is presented in Table 1**,** data on years of experience (Table 2**)**, data on economic sector where they belonged (Table 3**)**, data on procurement methods used by the respondents (Table 4**)** and time data on period of cost overrun experienced by them in executing construction projects **(**Table 5**).**

**Table 1:** Data profession of respondents.

Professional cadre of respondents	Frequency	Percentage
Architect	20	29.9
Builders	15	22.4
Engineers	15	22.39
Quantity Surveyor	10	14.9
Estate Surveyor	10	10.45
Total	70	100

**Table 2:** Data on respondents’ years of experience.

Years of experience	Frequency	Percentage
Above 10yrs	30	42.8
225-10yrs	20	28.6
1–5yrs	17	24.3
Missing data	3	4.3
Total	70	100

**Table 3:** Data on economic sector of the respondents.

Economy Sector	Frequency	Percentage
Private sector	47	67.1
Public sector	20	28.6
Missing data	3	4.3
Total	70	100

**Table 4:** Data of procurement methods used by the respondents.

Procurement methods	Frequency	Percentage
Traditional method	3	4.3
Project management	6	8.5
Direct labor	10	14.3
Design and build	20	28.6
Labor only contract	28	40.0
Missing data	3	4.3
Total	70	100

**Table 5:** Data on period of cost overrun experienced on projects.

No of Years	Frequency	Percentage
Above 2Yrs	0	0.00
1–2 years	2	2.9
6months-1year	21	30.0
Below 6months	39	55.7
Missing data	8	11.4
Total	70	100

Furthermore, severity index was used to obtain the ranks of cost-overrun determinants presented in Table 6. The data on impact of cost and time on project performance is shown in Table 7. The cost and time overrun survey information data on residential building projects are shown in Table 8 while the data is in agreement with those available in scientific literature as regards to the consequence of cost overrun.

**Table 6:** Data on determinants of cost overrun on construction projects.

Cost-overrun determinants	C.R. {5}	R {4}	J.R {3}	IRR {2}	V.R {1}	S.I %	R.K
Contractors Project inexperience	42	22	30	0	0	91.60	1
Inadequate planning	45	15	7	0	0	91.34	2
Inflation	42	20	5	0	0	91.00	3
Incessant variation order	44	16	6	1	0	90.70	4
Change in project design	43	17	7	0	0	90.70	4
Project complexity	42	20	3	2	0	90.40	6
Shortening of contract period	44	14	9	0	0	90.40	6
Fraudulent practices	42	18	7	0	0	90.40	6
Unstable economy	42	25	10	0	0	89.55	9
Inaccurate estimate	40	15	12	0	0	88.44	10
Overdesign	40	18	6	3	0	88.40	10
Project site location	35	25	5	1	1	88.05	12
Delay from employer	39	16	11	1	0	87.76	13
Force Majeure	30	25	11	1	0	85.10	16
Material Price fluctuations	30	18	19	0	0	83.30	14
Site conflicts	30	20	12	3	2	83.00	15
Poor workmanship	30	17	20	0	0	83.00	16
Inadequate financial provision	29	17	20	0	1	82.1	17
Contractors inefficiency	30	20	10	6	1	82.09	18
Unsteady material supply	30	15	20	2	0	81.80	19
Unpredictable weather condition	30	17	17	1	0	80.90	19
Breach of local regulation	25	22	11	8	1	79.10	20
Lack of executive capacity by employer	7	10	20	0	0	58.20	21

C.R= Completely relevant, J=Just relevant, IRR= Irrelevant, VR= Very Relevant, R.I= Relevant Index.

R.K= Ranking

**Table 7:** Data of impacts of time and cost on project performance.

Effects	R.A.I	Rank
Time overrun	0.796	1
Tied-up Capital	0.772	2
Loss of investment	0.756	3
Materials are effectively put to use	0.728	4
High tendency for the occurrence of dispute between the clients and contractors.	0.724	5
Project abandonment.	0.704	6
Excessive increase on the entire project cost.	0.656	7
Client's dis-satisfaction	0.640	8
Profit loss.	0.632	9
Consultant dissatisfaction	0.632	9
Payment delay	0.628	11
Good completion time	0.616	12
Maximized project profit	0.600	13
Reduced building component quality.	0.576	14
High level of material wastage	0.528	15

R.A.I= Relative Agreement Index

**Table 8:** Data of cost and time overrun survey information on residential building projects.

Assessment Statements	Architect	Builder	Structural	Quantity surveyor
I have been involved in a building project before	30%	40%	10%	10%
I have experienced extension in project delivery time	20%	50%	17%	13%
**Length Of Extension**				
1–6 months	0.89(i)	0.87(i)	0.85(ii)	0.86(i)
6–12 months	0.84(vi)	0.86(ii)	0.86(i)	0.83(ii)
12–18months	0.85(v)	0.85(iii)	0.82(iv)	0.82(iii)
18–24 months	0.87(iii)	0.85(iii)	0.84(iii)	0.81(iv)
More than 24 months	0.86(iv)	0.83(iv)	0.78(v)	0.82(iii)
**I have experienced cost overrun in a building project**				
				
Percentage Of Increase				
0–15%	0.78(vi)	0.65(vi)	0.66(vi)	0.65(vi)
15–30%	0.79(v)	0.76(iv)	0.73(v)	0.72(v)
30–45%	0.80(iv)	0.85(ii)	0.85(ii)	0.89(i)
45–60%	0.82(ii)	0.89(i)	0.87(i)	0.88(ii)
60–80%	0.81(iii)	0.71(v)	0.78(iii)	0.75(iv)
80% and above	0.83(i)	0.75(iii)	0.76(iv)	0.79(iii)

## 2 Experimental design, materials and methods

### 2.1 Data collection

Simple random sampling was used in the data collection through carefully structured questionnaire. A population size of seventy (70) was selected, and a total sample size of 59 respondents was used in this study, with questionnaire distributed to construction professionals. Variables pertaining to the above listed targets were identified and incorporated into questionnaires as the primary source of data. Some similar methods and contributions can be seen in [1], [2], [3], [4], [5], [6], [7], [8].

### 2.2 Data analysis

The data was collated and analysed, using mean item score ranking, percentages and the use of descriptive statistics. Cost overrun determinants were ranked in percentages using the severity index. The five-scale in the questionnaire forms the response variables which are mapped with the 23 cost overrun determinants to obtain the severity index. The five-scale response variables are listed with the assigned ranks: completely relevant (CR) is ranked 4, relevant is ranked 3, just relevant is ranked 2 and irrelevant is ranked 1. The summary is shown in Table 6.

Relative agreement index (RAI) is used to obtain the rank of 15 variables that determine the impact of cost and time on project performance. This is presented in Table 7.

The construction practitioners’ experiences on project cost overrun and duration were ranked distinctly and shown in Table 8. This enables for quick comparison and decision making.

The data composition is in agreement with those available in scientific literature as regards to the consequence of cost overrun. This is summarized in Table 9. The selected works relevant and similar can be found in [9], [10], [11], [12], [13], [14], [15], [16], [17], [18], [19], [20], [21].

**Table 9:** Data of consequences of cost overrun.

*Effects of Cost overrun.*	*Percentage*
Tying down of clients capital	80%
Company/firms liability to insolvency	50%
Liability of companies or firms to bad debt or bankruptcy	70%
Under-utilization of manpower resources	55%
Tendency for an increase project cost resulting from payments for idle and unproductive time arising out of contractors claims.	93%

Tendency for an increase project cost resulting from payments for idle and unproductive time	90%
Projects abandonment	60%
Under-utilization of plants and equipment	93%

## References

[1] Amusan L.M., Dosunmu D., Opeyemi J. (2017). Int. J. Mech. Eng. Technol..

[2] Odusami K.T., Olusanya O.O. (2000). Clients contribution to delays on completion cost of housing projects in Nigeria. Quant. Surv..

[3] Ogunsemi D.R., Jagboro G.O. (2006). Time-cost model for building projects in Nigeria. J. Constr. Manag. Econ..

[4] Okagbue H.I., Opanuga A.A., Adamu M.O., Ugwoke P.O., Obasi E.C.M., Eze G.A. (2017). Personal name in Igbo Culture: a dataset on randomly selected personal names and their statistical analysis. Data Brief..

[5] Bishop S.A., Owoloko E.A., Okagbue H.I., Oguntunde P.E., Odetunmibi O.A., Opanuga A.A. (2017). Survey datasets on the externalizing behaviors of primary school pupils and secondary school students in some selected schools in Ogun State, Nigeria. Data Brief..

[6] Iroham C.O., Okagbue H.I., Ogunkoya O.A., Owolabi J.D. (2017). Survey data on factors affecting negotiation of professional fees between Estate Valuers and their clients when the mortgage is financed by bank loan: a case study of mortgage valuations in Ikeja, Lagos State, Nigeria. Data Brief..

[7] Popoola S.I., Atayero A.A., Faruk N., Badejo J.A. (2018). Data on the key performance indicators for quality of service of GSM networks in Nigeria. Data Brief..

[8] Tunji-Olayeni P.F., Lawal O.P., Amusan L.M. (2013). Developing infrastructure in Nigeria: why is the cost so high?. Medit. J. Soc. Sci..

[9] Ashworth A. (1994). Cost Studies of Building.

[10] Bathurst P.E., Butler D.A. (1980). Building Cost Control Techniques and Economics.

[11] Dissanayaka S.M., Kumaransammy M.M. (1999). Comparing Contributory Time and Cost Performance in building projects. Build. Environ..

[12] Ferry D.J., Brandon P.S., Ferry J.D. (1998). Cost Planning of Building.

[13] Harris J.O., MacCaffer R. (2005). Modern Construction Management. Iowa State Press.

[14] Jagboro G.O. (1998). An evaluation of the factors that cause delays on construction project in Nigeria. Afr. J. Dev. Stud..

[15] Kerzner H.C. (1998). Project Management.

[16] Love P.E.D., Tse R.Y.C., Edwards D.J. (2005). Time-Cost relationship in Australian building construction projects. Niger. J. Constr. Manag..

[17] Majid M.Z.A. (1998). Factors of non-excusable delays that influence contractor's performance. J. Magt. Enginee.

[18] Mawdesley M., Askew W., O’Reilly M. (1997). Planning and Controlling Construction Projects.

[19] Mbachu J.K., Olaoye G.S. (1999). Analysis of major delay factors in building project execution. Niger. J. Constr. Manag..

[20] Pilcher R. (1992). Preicioles of Construction Management.

[21] Skitmore R.M., Ng S.T. (2003). Forecast model for actual construction time and cost. Build. Environ..

